# Mapping policies, regulations, and practice supports for medical office assistants in primary care: a scoping review

**DOI:** 10.1186/s12875-026-03222-8

**Published:** 2026-02-20

**Authors:** Jennifer Shuldiner, Apira Ragunathan, Jawairia Mohammed, Alan Katz, Meena Andiappan, David Barber, Amanda Condon, Gary Garber, Tara Kiran, Sylvia Hysong, Jill Schoon, Danielle Martin, Sabrina T. Wong, Noah Ivers

**Affiliations:** 1 Research and Innovation Institute, Women’s College Hospital, 76 Grenville St, Toronto, ON M5S 1B2 Canada; 2 Institute of Health Policy Management and Evaluation, University of Toronto, 155 College St 4Th Floor, Toronto, ON M5T 3M6 Canada; 3 Department of Family and Community Medicine, University of Toronto, 500 University Ave, 5Th Floor, Toronto, ON M5G 1V7 Canada; 4 University of Manitoba, Manitoba Centre for Health Policy, Winnipeg, MB R3E 3P5 Canada; 5 DeGroote School of Business, McMaster University, 1280 Main St W, Hamilton, ON L8S 4E8 Canada; 6 Queen’s University, 99 University Ave, Kingston, ON K7L 3N6 Canada; 7 University of Manitoba, 66 Chancellors Cir, Winnipeg, MB R3T 2N2 Canada; 8 Department of Medicine, Safe Medical Care Research Department, University of Ottawa, Roger Guindon Hall451 Smyth Rd, Ottawa, ON 2044K1H 8M5 Canada; 9 Canadian Medical Protective Association, 875 Carling Ave Suite 323, Ottawa, ON K1Y 4E3 Canada; 10 Department of Family and Community Medicine, St. Michael’s Hospital, Unity Health Toronto, 30 Bond Street, Toronto, ON M5B 1W8 Canada; 11 MAP, St. Michael’s Hospital, Unity Health Toronto, 209 Victoria St, Toronto, ON M5B 1T8 Canada; 12 Michael E. DeBakey VA Medical Center, Center for Innovations in Quality Effectiveness and Safety, 2002 Holcombe Boulevard, Houston, TX 77030 USA; 13 Durham Community Health Centre, 115 Grassmere Ave, Oshawa, ON L1H 3X6 Canada; 14 Department of Family and Community Medicine, Women’s College Hospital, 77 Grenville Street, Suite 252, Toronto, ON M5S 1B3 Canada; 15 University of British Columbia Centre for Health Services and Policy Research, 201-2206 East Mall, Vancouver, BC V6T 1Z3 Canada; 16 School of Nursing, 2211 Wesbrook Mall T201, Vancouver, BC V6T 2B5 Canada

**Keywords:** Medical Office Assistant, Primary Care, Health policy

## Abstract

**Importance:**

Medical Office Assistants (MOAs) are non-clinicians who carry out critical tasks in primary care settings. Despite their central roles as the first point of contact for patients or at the front desk, there are no reviews of policies, supports or interventions that could help support MOAs within complex primary care clinics.

**Objective:**

We systematically scoped the literature to identify interventions, regulations, policies, practice supports, or resources targeting MOAs in primary care.

**Evidence review:**

Searches were conducted in Pubmed, EMBASE, Web of Science, and grey literature sources (Google, Google Scholar, and Duckduckgo), for items set in high-income countries and reported in English or French, from January 2000 to December 2024. We additionally searched for references for all articles through Scopus. Articles, reports, papers, or other online materials or articles were included if they reported anything about supporting MOAs in primary care clinics. Data analysis involved descriptive numerical summaries and content analysis.

**Findings:**

Sixty articles were included, covering team building or reconfiguration of the team (18/60; 30%), education/counselling/health coaching (15/60; 25%), navigator or care management of patients (10/60; 17%), training or credentials for MOAs (8/60; 13%), screening activities (6/60; 10%), and advanced rooming (3/60; 5%). Articles were primarily set in the United States (47/60; 78%). Workforce well-being was the most common positive outcome (26/60; 43%). Equity outcomes were rarely reported (5/60; 8%). Commonly identified barriers to implementing interventions included time and resource constraints, staffing challenges, inadequate training, and lack of provider buy-in. Involving MOAs in planning, offering role flexibility, and fostering leadership support were important for success. Furthermore, strong leadership, collaborative relationships, and fair compensation were key components of an environment conducive to change.

**Conclusion and relevance:**

This review reveals gaps in supporting MOAs as members of the primary care team. Most of the literature focuses on clinic-level changes, with limited evidence on MOA training and/or career growth. Given their strong impact on primary care access and experience for patients, more focus on MOAs in health system reform is warranted.

**Supplementary Information:**

The online version contains supplementary material available at 10.1186/s12875-026-03222-8.

## Background

Medical office assistants (MOAs) are core members of the health care workforce. We use the term MOA to describe non-clinicians who complete a range of duties [1–3]. In addition to administrative tasks like scheduling appointments, managing patient records, billing, and ensuring smooth office operations [1, 4], many MOAs conduct clinically-adjacent tasks such as triaging patients, obtaining vital signs, reporting results of investigations to patients, or administering screening questionnaires [5–8]. They also serve as a communication bridge between patients and clinicians. In primary care settings, they represent the front-door to the entire health system, and routinely conduct crucial administrative, communicative, problem-solving, and decision-making tasks [3, 9, 10]. MOAs in primary care often having deep insights about patients’ personal and family histories [5]. Despite this, their health-related training is often minimal [2].

The role of MOAs and their titles differs across jurisdictions. For example, in the UK, MOAs focus more on administrative rather than clinically-adjacent tasks [11] and have mandated online education in triage and patient safety [12]. In Australia and Canada, MOAs perform both administrative and clinically-adjacent tasks, and some have training through accredited courses, though the scope of clinical duties varies widely. In the US, medical receptionists are focused on administrative tasks while medical assistants have a range of clinical-adjacent responsibilities, including rooming, vital signs and assisting with procedures, with certification typically required [13].

The primary care sector has experienced a substantial increase in workload as the population ages and the complexity of care increases [14]. Policy documents on the challenges and opportunities facing primary care highlight the need to realize the full potential of both clinical and non-clinical staff [15, 16] including MOAs. Unfortunately, MOAs face many challenges related to professional development and task performance. MOAs in primary care are typically not licensed and rarely unionized in the primary care environment [17]. Relatedly, they have large variations in skills and training, often with no clear pathways for continuous education and career-related advancement [18]. The wide variation in tasks performed by primary care MOAs reflects the need for flexibility and adaptation to local needs, but to achieve the full potential of their role, some health system partners have expressed interest in pursuing policies to enable system-wide efforts at standardizing or optimizing their skill set [19, 20].

The objectives of this scoping review are to systematically scope the relevant literature on the work of MOAs in high-income countries, and identify regulations, policy levers, practice supports or resources that support the quintuple aim (i.e., population health, patient care, workforce well-being, efficiency, and equity) [21, 22]. By identifying gaps and opportunities in the current systems, this review seeks to inform strategies that could optimize the role of MOAs, ensuring they are better equipped to meet the demands of modern primary care.

## Methods

We conducted a scoping review of regulations, policy levers, and practice supports for MOAs, reported results following the PRISMA (Preferred Reporting Items for Systematic Reviews and Meta-Analyses) extension for scoping reviews [23] and registered our protocol in the Open Science Framework database [24]. Our methodology is based on Arksey and O'Malley's [25] approach and Levac et al.'s [26] methodological enhancement and featured the following steps: (1) identifying the research question; (2) identifying relevant articles; (3) selecting articles; (4) charting the data; (5) collating, summarizing and reporting the results and (6) consulting with relevant stakeholders. We consulted with a Scholarly Communications & Health Sciences Librarian to design and refine the search strategy for this review.

We assembled an interdisciplinary team across Canada with expertise in implementation science, organizational theory, qualitative methods, biostatistics, primary care reform, health policy, and health services research (*N* = 27). The team also includes an MOA advisory group that provides input on methods, results and analysis through meetings and reviewing documents. Throughout the study, the team met regularly to discuss the protocol, review emerging results, and interpret findings, ensuring a collaborative and rigorous approach to the research. No formal methodological quality or risk-of-bias appraisal was undertaken; the review synthesized reported findings as described. We also recorded whether studies reported funding.

### Stage 1: identifying the research question and consulting with key groups

Based on initial exploratory research and a meeting with the research team and key stakeholders, we developed our research questions and submitted the protocol to the Open Science Network:

1. What regulations, if any, exist that govern the scope of work, training requirements, and payment policies for primary care MOAs in different jurisdictions across high-income countries as defined by the World Bank?
2. What policy levers, practice supports, educational programs, or knowledge translation resources exist to support high performance amongst primary care MOAs?
3. What evidence exists related to the effects of these supports on the quintuple aim (population health, patient care, provider experience, cost-effectiveness, and equity)?

### Stage 2: Identifying relevant articles- Search strategy and information sources

#### Eligibility criteria

We identified articles through a search of electronic databases and grey literature. Based on the initial exploratory research we used the following eligibility criteria:

- Type of publication: journal articles, reports or papers, conference proceedings
- Study design: Any study design, report or commentary
- Time frame: Published in 2000 or later (to account for changes since the introduction of electronic records)
- Language: English or French
- Location: Not in a low-or-middle-income country (as defined by the World Bank) to find evidence most likely to be relevant to the Canadian context
- Study population: medical office assistants (e.g., medical office assistant, receptionist, medical assistant, front office staff) working in office-based primary care practices (e.g., family physician, general care)
- Types of intervention: regulations, policy levers, practice supports or resources implemented at scale and or across jurisdiction

#### Information sources

The search strategy consisted of the two concepts of ‘Medical Office Assistant’ and ‘primary care’. It was run in the following databases: MEDLINE on Ovid, EMBASE on Ovid, CINAHL on EBSCO, APA PsycInfo on Ovid on September 11, 2023 using database specific syntax to search a combination of text words, keywords and subject headings. The search strategy can be found in Appendix 1. For the concept of primary care, Wetzels et al. (2007) search strategy [27] and Flinders Primary Care search filters [28] were consulted and adapted. A date limit of 2000 to present was put on the search strategies. Reference tracking backwards and forwards using Scopus was conducted on all items that were included and any articles not retrieved on Scopus are summarized in Appendix 2.

#### Grey literature search

For the concept of primary care, we consulted and adapted Wetzels search strategy and Flinders Primary Care filters. We underwent a multi-step grey literature strategy [29], with details found in (Appendix 3a-e):

A) Grey Literature Databases included: We searched ProQuest Thesis Dissertations, Open Grey (system for information on grey literature in Europe), the New York Academy of Medicine Grey Literature Report.
B) Search Engine Searches: We conducted custom Google Scholar searches with the same terms we used in our academic literature search. We set limits for high-income countries as defined by the World Bank. We reviewed the results 10 pages past the last relevant site found from the Google Scholar search. A grey literature search was also conducted in Duck Duck Go, using different search strings including various terms under each concept of ‘MOA’, ‘supports’ and ‘primary care’ (Appendix 3e).
C) Targeted Website Searches: We browsed (using a variety of identified words) the websites of organizations (government, health organizations, NGOs, universities, research centers, community organizations, advocacy groups, and academic departments) that publish documents relevant to the research question.
D) Targeted Website Search for MOA Programs: An advanced Google search was conducted for each of the 38 OECD countries from July to August 2024 to identify programs offering medical office job training (Appendix 4), using search terms developed from a preliminary search (Appendix 1). The search reviewed the first five pages of results to find relevant certifications, diplomas, standardized learning modules, and training programs, excluding job postings, handbooks, foreign exchange programs, medical volunteering, and government sites. Only programs with publicly available information were included, ranked by Google’s relevancy. For countries with no results, job terminology was verified, and searches repeated.

### Stage 3: study screening

Citations identified through the bibliographic databases were deduplicated using Covidence. Two team members independently screened the titles and abstracts to assess eligibility. The review process consisted of two levels of screening: (1) a title and abstract review and (2) full-text review. For the first level of screening, two researchers (AR and JM) independently screened the title and abstract of all retrieved citations for potential eligibility against a set of minimum inclusion criteria. Articles deemed relevant by both reviewers were included in the full-text review. In the second step, the two researchers (AR and JM) each independently assessed the full-text articles to determine if they meet the inclusion/exclusion criteria and reasons for exclusion were recorded at this stage. Conflicts were resolved through discussion or with the input of a third reviewer (JS and NI), as needed.

### Stage 4: data collection

We used excel for extracting study characteristics, which included: article details (article title, author(s), year of publication, country of study, type of document/study, objectives, methods/approach, participants, MOA title used in study), details of the support/policy/intervention (name, brief description, key findings/learnings, areas of influence, contextual factors, barriers and facilitators), and outcomes organized by their impact on the quintuple realm (outcomes related to patient/care experience, workforce well-being, population health, cost of care/efficiency and equity).

Data abstraction was conducted by two members independently (AR and JM). Each reviewer's independent abstracted data were compared, and discrepancies were discussed with a third reviewer (JS). This process was to confirm consistency between the reviewers. Data was compiled in an excel spreadsheet.

### Stage 5: data summary and synthesis of results

Upon completion of data collection, we organized the data into a priori categories of policies, regulations, and practice supports, developed by the team based on the literature and consultation with our advisory team. These categories were 1) tasks, split into 5 subcategories of education/health coaching, navigator/care management, panel management, screening activities and advanced rooming 2) training and/or credentials and 3) team building or reconfiguration of the team. Two researchers categorized each intervention and came together to discuss discrepancies (AR and JM). A third researcher resolved any conflicts (JS).

Each item found was reported by category with their outcomes organized by the quintuple aim (improving population health, enhancing the care experience, reducing costs, workforce well-being and safety, equity) [21, 22]. For equity, we assessed whether the explicit focus of the project described was to improve care for a marginalized group, or if this was assessed as a secondary feature, such as through sub-group analyses, as we have done in prior reviews [30]. Our findings provide an overview of the research rather than an assessment of the quality of individual articles. The study team met to collate and summarize study results [25], and then to compile and organize results into category-specific tables that relate to our research questions. In this way, our scoping review provides a synthesis of supports, interventions and potential policy levers to help MOAs achieve their full potential.

### Stage 6: consultation

As per Level et al. [26] we consulted with stakeholders in Canada, and research team partners in Canada, United States, and the United Kingdom who provided insights on the scoping review protocol. Stakeholders, in particular our MOA advisory board, were engaged throughout the study and acted as consultants. Specifically, they provided input regarding the search strategy and grey literature search to incorporate the MOA voice in capturing what is important.

## Results

### Search results

**Fig. 1:**
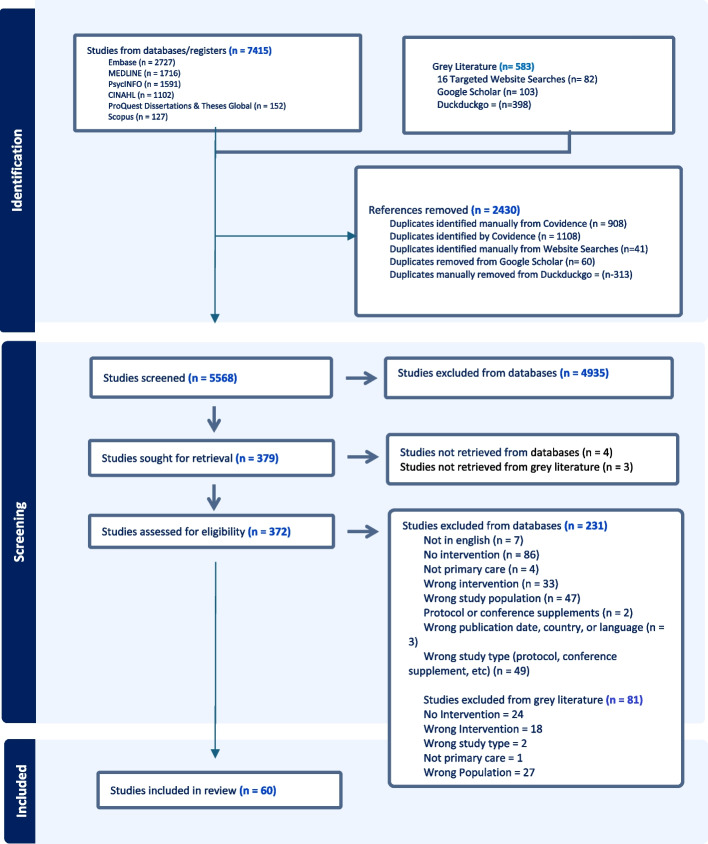
PRISMA, Study identification and selection

The search identified 7415 records from databases/registers that were imported into Covidence. The grey literature search found 583 articles to be considered for eligibility and were documented on a Word Document. A total of 2430 duplicates were identified from both the grey literature, supplementary and database searches, either manually by the team or automatically from Covidence (Fig. 1). 5568 citations entered title abstract screening, 379 full texts were retrieved and assessed for eligibility and 60 articles were included (42 from databases searches, 18 from grey literature). Reference tracking backwards and forwards using Scopus was conducted on the 60 items included in the review. Of the 60 articles included in the review, Scopus could not identify two items (Appendix 2). 126 citations were retrieved from reference tracking in Scopus, exported into Covidence and screened, and all were excluded.

### Description of included articles

Most articles took place in the USA (47 articles), followed by the UK (6 articles). Articles also took place in Germany (2), Canada (3), Switzerland (1) and Australia (1). We found academic manuscripts or theses (44), reports (11), commentaries (2), toolkits (2), and a book (1), but no policy papers, as demonstrated in Table 1. Participants commonly used the titles Medical Assistants (42), Medical Office Assistant (5), Receptionist (5), others (5), Front Office Staff (2), Office Staff (2), and Manager (2). A few articles used participants with various MOA roles (e.g. interviewing managers and office staff).

**Table 1:** Description of the type of sources included

Source	Number of items
Academic publications (manuscripts or theses)	44
Reports	11
Commentaries	2
Toolkits	2
Book	1

Across the 60 articles, we found policy levers and practice supports among most categories, as seen in Table 2 below.

**Table 2:** Types of policy levers and practice supports identified for Medical Office Assistant in primary care

Type	Subtype	Definition	Number of results
Patient Support and Care Coordination	Education/Counselling/Health Coaching	providing advice to patients with previously diagnosed conditions on self-management	15
	Navigator/Care Management	coordinating services or appointment patients for patients, or advice about coordinating services or appointment for patients, or providing advice about available community services	10
	Panel Management	find patients due for screening – using data to identify patients that are due for something	0
	Screening activities	mental health, smoking, alcohol; this includes data collection from patients, can be on the phone, in person, or via a survey	6
	Advanced rooming	Taking vitals, support provider in rooms	3
Training or credentials for Medical Office Assistant		Training courses, education, career ladders, credentials for MOAs	8
Team building or reconfiguration of the team		Changing roles, new team members, task/role blurring, change of scope	18

From these, 51 articles reported quintuple aim outcomes (Table 3).

**Table 3:** The number of studies organized by quintuple aim categories reported*

Types of policy levers and/or practice supports (# of included articles)	Subtypes of policy levers and/or practice supports (# of included articles)	Quintuple Aim Categories				
		Patient Care	Workforce Well-being	Population Health	Cost of Care/ Efficiency	Equity
Patient Support and Care Coordination (34)	Education, Counselling, Health Coaching (15)	9	11	6	4	2
	Navigator/Care Management (10)	5	7	4	4	0
	Panel Management (0)	0	0	0	0	0
	Screening activities (6)	2	5	3	3	2
	Advanced Rooming (3)	1	3	2	1	0
Training or credentials for MOA (8)		3	6	2	2	1
Team building or reconfiguration of the team (18)		7	14	3	7	0
	Total	27	46	20	21	5

^*^9 articles had no outcomes

Table 4 reports outcomes organized by the quintuple aim framework. The full details of the outcomes of each article can be found in Appendix 6 (Tables A1-A5). No payment policies were identified among the articles included.

**Table 4:**
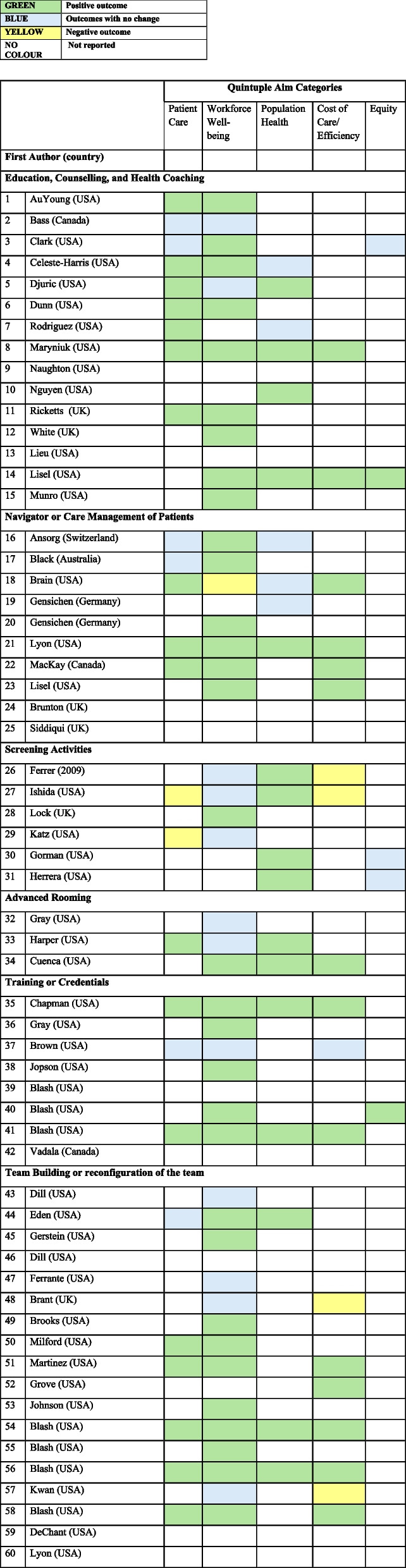
Summary of quintuple aim categories for policies, regulations or practice supports*

## Section 1: patient support and care coordination

### Education, counselling and health coaching

As demonstrated in Tables 3 and 4, 15 articles focused on MOAs providing education, counselling or health coaching to patients. Eight articles focused on education and counselling and six on health coaching. 12 articles were published in the United States, two in the United Kingdom and one in Canada. Topics in the education and counselling interventions included tobacco usage, diabetes, depression screening, suicide assessment, routine testing (height, weight, blood pressure, etc.), mental health knowledge, using decision-making aids on contraceptive methods and general approaches to helping MOAs empathize and tailor interactions with patients. In the six health coaching articles, MOAs supported management of obesity [31], diabetes [32–34], sleep, diet, physical activity, and behavior change goals [35] and chlamydia [36]. 2 articles had no reported outcomes [37, 38].

Eight interventions reported outcomes related to patient care. Three interventions demonstrated positive patient care outcomes (e.g. motivation to achieve health goals due to increased role of MOAs in their care) [31], satisfaction with care [35] and confidence in MOAs [33]. One intervention found that patients reported more positive experiences when engaging with an MOA in an expanded health coaching role compared to interactions with a community health worker [39]. Four interventions found increased documentation of patients at risk [40], behavioral health screening [41], chronic care provided by MOAs [39] and frequency of MOAs speaking with patients [42]. An intervention that assigned MOAs the responsibility of promoting diabetes educational resources, lead to increased patient engagement in clinic-based diabetes care [42].

Ten interventions impacted workforce well-being. Staff confidence in their roles, responsibilities, and knowledge improved in four interventions [29, 33, 38, 40]*.* Three interventions found increased physician satisfaction with MOA’s intervention work [30, 32, 39]*.* One intervention found positive career impacts, such as higher salaries and increased opportunities for MOAs [31]. Another intervention found that MOA communication skills improved [43]. Knowledge and awareness regarding depression increased in one study [40]. Another study found MOAs were generally enthusiastic about their new health coaching role [28].

Population health was impacted by 5 interventions. Patient’s quality of life and optimism towards their care improved when MOAs assisted with their diabetes care [33]. Several articles found positive uptake of clinical outcomes such as increased testing of blood pressure and cholesterol [34, 35, 42, 44].

One article impacted cost of care and efficiency, where there was an increase in organizational efficiency [34]. One intervention impacted equity, as they increased opportunities for bilingual MOAs who could support patients in need of culturally appropriate services [34].

### Navigator and care management

There were ten articles spanning six countries: Switzerland (1), Australia (1), the United States (3), Germany (2), the United Kingdom (2) and Canada (1). Interventions included training MOAs to provide routine diabetic care [45–47], conducting initial counseling and screening for patients with elevated body mass index (BMI) [46, 48], implementing a protocol-based care management for patients at risk of rehospitalization for mental health care [49, 50], redefining MOA responsibilities in chronic disease management, increased provider to MOA ratio for patient visits [51], care coordination for elderly patients [52] and signposting [53, 54]. Two papers did not report outcomes [53, 54].

In terms of patient care, two interventions reported positive effects on patient satisfaction [46, 48], while one observed a negative impact [45]. Staff and provider communication with patients improved in two interventions [46, 51].

Six interventions reported positive impacts on workforce well-being [45–47, 49, 51, 52]. By creating clinical roles to care for diabetic patients, staff felt roles were clearly defined and they were working more efficiently [45, 46]. Interventions resulted in less provider burnout [51], improved job satisfaction and confidence [49], as well as increased willingness to participate in quality improvement activities [46]. Lower MOA turnover was also found [52]. Task shifting with routine diabetic care from physician to MOAs reduced physician burnout and the intervention received positive feedback from physicians [45]. One intervention had a negative impact on workforce well-being as time constraints and staffing challenges were measured [48].

Regarding cost of care and efficiency, four interventions reported positive changes [46, 48, 51, 52]. Brain et al. found that by redefining responsibilities for MOAs to care for chronic disease management, emergency department visits, urgent care visits, and hospital admissions reduced [48]. Three interventions found increased visit volumes [46, 51, 52] alongside decreased provider overtime hours [51] and decreased cost [46, 52].

### Screening activities

There were five MOA-led screening programs [55–59] and one was a care coordinator role developed to increase screening with MOA involvement [60]. Five were in the USA, while one was in the UK.

Three articles found a negative impact on patient care experience. One study found that patients did not understand the rationale behind the intervention, in which automated messaging was used to promote screening [60], and another found that patients of MOAs were significantly less likely than patients of registered nurses to be identified for screening [57]. Another article found that resources provided had limited patient utility (e.g., outdated or unhelpful to patients) [59].

Regarding workforce well-being, one study found that MOA’s attitudes towards their involvement in the intervention changed positively during implementation [56]. Another found that MOAs were willing to learn and take on new tasks, however they also found that MOAs had decreased satisfaction with their role [57]. One article found that the intervention allowed MOAs to practice more holistic care, such as guiding patients to seek help for local community services (housing, daycares, etc.), however authors also reported that it had negative impacts on workforce well-being as there was confusion from MOAs about the intervention which impacted workflow [59].

With respect to population health, two articles found an increase in patient screening [55, 58] and the other found an increase in patients completing advanced care planning discussions for colorectal cancer [60].

Two articles measured a negative impact on cost of care and efficiency. One found low fidelity of the intervention as the practice struggled with adoption due to pressure to keep up with patient flow [55], and the other found an increase in wait time and time spent with staff [60]. As for equity outcomes, one study found that screening rates were higher for Black/African adults when completed by MOAs compared to physicians [58] and another article described that intervention was offered in multiple languages for families ensuring that language does not prevent access [59].

### Advanced rooming

Three articles involved interventions where MOAs supported advanced rooming initiatives, one in which MOAs became flow managers [61], another in which the MOA role was expanded [62], and lastly an MOA supported the doctor during a patient visit [63]. One article did not report outcomes [61].

Expanding MOA roles positively impacted workforce well-being, increasing physician confidence and satisfaction with MOAs [62] while also enhancing MOAs' skill development [63]. Health outcomes improved as well with an increase in mammograms [62] and other cancer screening [63]. Clinic efficiency improved, visits were shorter, which in turn made clinic flow smoother [62]. Another study reported lower wait times for patients and more efficient visits (e.g. better communication and quicker results provided) [63].

## Training and credentials

Seven articles and 1 book discussed training and credentials for MOAs, all set in the USA [13, 64–70]. Regarding patient care experience, articles reported increased patient satisfaction of MOAs’ role in their care [13, 69], improved patient perceptions of MOAs [65], and patient comfort discussing mental health [65].

Regarding workforce wellbeing, four training programs found an increased in staff satisfaction with their work and/or with the program [13, 64, 68, 69]. Others reported increased staff engagement, motivation and/or confidence in delivering the intervention [13, 64–66]. One study found that career development opportunities increased job satisfaction and engagement, reduced MOA turnover, improved teamwork, collaboration and quality of care delivery and used apprenticeships to upskill MOAs into new roles [66]. Recruitment and retention of MOAs increased in two articles [68, 69].

Regarding population health outcomes, one study found that screening of asthma and blood testing increased [13], and another found increased immunization and colorectal screening rates [69]. Regarding cost of care and efficiency, one study found a positive impact as patient visit time, emergency department visits, urgent care visits, and hospital admissions all decreased, while returned phone calls to patients increased [13]. Another study found less patients visits with primary care, ER, hospital admissions, and specialty care [69]. Regarding equity, one intervention reported that MOA’s language skills enabled them to help non-English speaking patients [68].

## Team building or reconfiguration of the team

Eighteen articles focused on team building initiatives or reconfiguration of the team in primary care clinics. 17 articles were set in the USA, and one in the UK. Models included: building a career ladder [71, 72], team-based primary care models [73–75], patient-centered medical home (PCMH) models [19, 76–78], alternatives to face-to-face consultation [79], team reconfiguration [80, 81], work performance tool [82], quality improvement interventions [83], a quality assurance committee conducting evaluation [84], strategies for staff retention and recruitment [85], and a toolkit to build relationships in clinics [86]. 3 articles had no reported outcomes [19, 85, 86].

Regarding patient care, patient satisfaction increased in 4 articles [77, 78, 82, 84], and patient gratitude towards the care given increased in one study [74]. One study found that patients did not use telephone consultations because MOAs did not promote them, which was attributed to their lack of involvement in planning, training, and acceptability of the new approach [79].

Regarding workforce well-being, workforce satisfaction for staff and physicians increased across eight interventions, [72, 74, 76–78, 80, 82, 84]. Two interventions found an increase in wages and career opportunities [71, 77]. Four interventions found an increase in recruitment and retention of MOAs [72, 77, 78, 84]. Increased MOA fulfillment and tolerance for workload was also reported [73]. One study found improved teamwork and increased career ladder opportunities [77].

Population health was impacted in two articles. Implementing a quality assurance committee to improve chronic care access and efficiency (such as diabetes care and immunizations) resulted in increased diabetic care follow-ups and monitoring, children's immunization, and cardiovascular disease diagnoses [19]. In the second study, the ratio of MOA to physician was increased and MOAs took on physician tasks to improve efficiency. This resulted in an increase in vaccinations, diabetes monitoring, and breast screening [81].

Regarding cost of care/efficiency, one study was found to be cost-effective for the practice [79], and another experienced staffing challenges [81]. Two articles found that interventions allowed more time for staff to complete work [82, 87]. Two articles found improvements in clinic efficiency, such as a decrease in abandoned calls [72] and shorter wait times [84]. Finally, a clinic found that increasing the MOA to provider ratio, led to staff cost decrease and a 50% drop in patient no-shows [78].

## Reported barriers and facilitators

We thematically organized barriers and facilitators from across articles (Table 5). Barriers to implementing interventions included time and resource constraints [13, 19, 31, 32, 36, 38, 39, 43, 45, 47, 48, 55, 61, 66–69, 76, 78, 81]. These included overestimating clinic capacity [31, 43, 45, 48, 55, 76] and difficulties in recruiting [ 19, 38, 39, 81] and retaining trained staff [32, 61, 76]. Inadequate training and a lack of provider buy-in complicated implementation [13, 38, 68]. Some MOAs felt unmotivated and unclear about the interventions' purpose [13, 61]. MOAs were also often uninvolved in the planning and implementation of the intervention, inhibiting buy-in [36, 55]. Also, providers resisted collaboration, distrusted MOA skills, and were uneasy with role changes [32, 37, 55, 61]. Resistance to adapting to new workflows and worries about legal compliance hindered organizational change [51, 56]. Finally, insufficient compensation for additional responsibilities was also reported [19, 51, 56, 76, 85].

**Table 5:** Reported barriers and facilitators of intervention or program implementation

Barriers	Facilitators
*Time and Resources constraints* • Overestimation of clinic capacity and difficult workload • Recruiting highly trained staff • MOA burnout and turn over • Maintaining old roles (e.g. running front desk) while implementing new ones *Training and Knowledge* • Inadequate resources dedicated to training • Allocating MOA time to attend training, and providing coverage for training • Role unpreparedness due to lack of training • Buy in from providers that training was required *Motivation and Acceptance* • Retention of trained MOAs • Some MOAs lacked interest and education background • Unclear purpose of the interventions • Reluctance to take on new tasks, more work, or work on a team • Job dissatisfaction for the MOAs • Some MOAs were not in favor of blurred roles • Reluctance of providers (changing clinic structure, presence of MAs in exam room) and distrust with MOA skills • Role perception: new MOA role not positively received by GPs and staff • Lack of engagement of PCP to integrating new MOA roles • Staff seen as lower status compared to clinicians *Complex Patient Interactions* • Complexity of population (e.g., language, SES) • Patient's issues were not in the scope of MAs • MAs felt hypocritical delivering messages at odds with their own behavior • Discomfort with engaging patients in mental health discussions *Collaboration and support* • Lack of an internal champion • Resistance from providers and administrative staff who did not think MOAs were up to the tasks • MOAs did not feel a part of the planning process • MA's felt lack of confidence • lack of MOA recognition *Organizational change and adaptation challenges* • Difficult for practices to adapt to the innovation • Lack of team huddles • No head or director to give feedback • Lack of planning and coordination to integrate new MOA roles *MA Career Opportunities* • Lack of clarity of MA scope • Lack of stackable credentials • Lack of career mobility in clinics e.g. no promotion opportunities, no compensation for added work) *Legality, Policies, Systems* • Compliance, legal, billing, and other internal regulatory oversight of the new delegated order protocols • Concerns about shared documentation in the EHR • Did not have permission to order screenings • Lack of compensation for more work	*Champions and Leaders* • Institutional Support: Clinic leaders and program champions increased enthusiasm • The quality improvement lead facilitated biweekly implementation meetings • Use talented MOAs as teachers *Training and Skills* • Staff training • Creating formal partnerships with MA training institutions to provide “externship” + opportunities • Limit training sessions to shorter times • Communicating the training to physicians • Sense of control and efficacy over work *Patient Interactions and Relationships* • Patient-provider relationship • One-on-one supports provided by MOAs to increase patient engagement • Increased involvement with patients *Accessibility* • Intervention offered in English and Spanish • Connecting with community resources outside the practices enhanced capacity *MOA engagement and role redevelopment* • Input from medical assistants and desire to take on these additional responsibilities • Flexibility to switch between traditional and new roles • Working with human resource to establish a career ladder prior to implementing changes *Time and Resources* • Time and opportunity to explore the workflow decreased frustration • Top leadership buy-in • Clinics from large health systems are well-resourced and supported by human resources workforce development *Office Relationships* • Engaging providers & nurses in training to embrace change • Align vision and goals with providers and MAs • Personal connections with colleagues and having psychological safety • Ability to give input and be respected • A sense of trust/safety in a positive work environment *Office Structure* • Detailed protocols and standing orders • ability to make structural changes *Compensation, Benefits* • Designing and distributing appropriate rewards for workers as they increase their skill levels • Compensation for extra effort *Other* • Developing “bright spots” to streamline implementation before implementing system-wide • Mix MAs from different sites to enhance community and increase participation

Key to the intervention's success was strong support from clinic leaders and program champions [19, 31, 62, 71, 74]. Also. training modules and partnerships with training institutions prepared MOAs for their new roles [13, 19, 31, 76, 80]. Collaborative relationships among clinic staff and providers, and engagement with MOAs encouraged a shared vision [37]. Some interventions also offered services in English and Spanish, and connection with community resources [19, 43, 55]. This improved accessibility and capacity. Input from MOAs, along with their flexibility to transition between traditional and new roles, was crucial, as well as having a clear career ladder [13, 19, 71, 81]. Additionally, top leadership buy-in fostered an environment conducive to change [13]. Engaging MOAs, providers, and nurses in training created alignment in vision and goals [13, 32, 38, 69].

## MOA training programs by country

Relevant and country-specific MOA related programs were found for 23 out of 38 countries. While searches for the UK and US generated higher number of results, more programs in these countries were online programs and lacked practicum or internship opportunities.

Overall, while there is a core set of skills common to most countries' programs, there are also notable differences (Appendix 5). Programs that covered all skill sets were found in Australia, Canada, Japan, Norway, Germany, and New Zealand. The most common subject area across all countries’ programs was administrative skills, which were found in programs in 19 countries. The second most common course subject was medical terminology, which was found in programs in 10 countries. EMR specific knowledge was found in 9 countries and computer skills were found in 15 countries’ programs.

The most common length of programs was one year, with the shortest online program being 3 h (United Kingdom) and the longest being 3-year in-person programs (Denmark, Germany). In general, in-person courses tend to be longer, often ranging from several months to years, while online courses are typically shorter, ranging from a few weeks to several months.

The cost of programs also highly varied and was often not included on program websites. From the public information gathered, the most expensive programs were in Canada ($9,300 USD) and Australia ($5,100 USD). Some programs were paid for through practicum placements, so students did not need to provide a course or registration fee (Germany & Norway).

## Discussion

Despite the critical role of MOAs in primary care, this is the first review of policies, regulations, and practice supports to help ensure that MOAs can perform an evolving range of tasks in their complex primary care environment. Most articles in our review focused on clinic-level changes – from shifting team structures, to creating new roles, and developing efficient workflows. There were only a few resources identified regarding training to support MOAs.

The two largest categories of articles focused on patient support and care coordination (e.g., health coaching, care navigator, screening activities, advanced rooming), and team building and reconfiguration. Few articles focused on MOA training and credentials, indicating a need for more evidence to support MOA learning and approaches to expanding MOA capabilities beyond their current administrative duties.

The most reported outcomes related to workforce well-being, which included MOA job satisfaction, engagement, motivation, leadership, recruitment, and retention. This aligns with descriptive articles that have identified career aspirations for MOAs who were interested in growing in their roles [88]. Population health outcomes such as increased screening and quality of life were most reported in education, counselling and health coaching interventions. Cost of care/efficiency outcomes were commonly reported in articles that involved team building and reconfiguration of the team interventions.

Only five equity-related outcomes were measured across all articles, highlighting a gap in research and policy on how MOAs can support patients facing structural barriers to care. These articles focused on language barriers and equitable healthcare services for racialized communities, suggesting the potential for MOAs to enhance equitable patient care—for example, by employing bilingual MOAs to improve communication and access.

Regarding education and counselling, positive outcomes were found mainly in patient care, workforce wellbeing and population health. Team building and reconfiguration had the greatest impact on workforce well-being, likely due to MOAs strong desire to help patients [89], which these interventions actively supported.

Barriers to implementing interventions included time and resource limitations, challenges in staffing, inadequate training, lack of preparedness, and lack of provider buy-in. MOAs were often uninvolved in planning, leading to low motivation, while providers resisted collaboration and role changes. Complexities within health systems, including legal restrictions on scope of work, as well as lack of career advancement opportunities, also hindered interventions. Facilitators included strong leadership, training support, and collaborative relationships, which fostered a shared vision among clinic staff. MOA input, time and resources, role flexibility, fair compensation and benefits, and leadership buy-in were key to creating an environment conducive to change. These success factors align with descriptive articles that suggest MOAs derive satisfaction from aspects such as task variety, assisting patients, and fostering positive relationships with colleagues [89].

Most health professions can be understood through their licensure, legal requirements, and professional organizations, but the work scope of MOAs in most jurisdictions is only loosely regulated. The proportion of MOAs with formal training is difficult to determine as they are not registered with a licensing body [4] and training is often “on-the job” [90]. Our search of MOA programs found substantial variability in the availability, content, structure, and focus areas. The findings highlighted differences in program duration, from 3-h online courses to 3-year in-person programs, as well as common skill areas, and the prevalence of online versus in-person learning. While certain core competencies like administrative skills and medical terminology were consistent, there was variability regarding areas such as ethics and privacy and EMR-specific knowledge. Additionally, there was a wide variation in program costs, with some of the highest fees in Canada and Australia.

Expanding the scope of practice and training for MOAs has the potential to help address health system strain by improving access to care, enhancing efficiency, assisting patients in navigating complex services, and strengthening triage and intake processes. Leveraging the skills and experience of MOAs within team-based models of care may contribute to a more effective and patient-centred health care system, and support progress toward achieving the quintuple aim.

### Limitations

This scoping review had a few limitations. First, we could not access non-academic resources and documents that were not publicly available. This review was restricted to articles published in English and French, so relevant resources and articles in other languages, were excluded. This language limitation may have resulted in gaps in the coverage of programs and initiatives from non-English/French speaking regions. No formal methodological quality or risk-of-bias appraisal was undertaken. Findings may be influenced by favorable response bias common in literature on care improvement initiatives as well as publication bias toward positive results; together these factors may overstate benefits and underreport neutral or negative findings. Finally, there was difficulty with the academic literature search given a lack of shared terminology for MOAs.

## Conclusions

This review highlights evidence gaps regarding policies and structured supports to enhance the work of MOAs in primary care. Most of the evidence identified focuses on clinic-level changes, with limited attention to MOA training or career advancement. Despite their presence in all health care teams and strong potential impact on primary care access and experience for patients, MOAs remain under-recognized in broader health system policies and related research.

## Supplementary Information

Supplementary Material 1.

## Data Availability

All data and materials can be found in our Appendixes.

## Abbreviations

MOA: Medical Office Assistant
PRISMA: Preferred Reporting Items for Systematic Reviews and Meta-Analyses
Medical Assistant: Medical Assistant
