# Supplementary material for: Mapping policies, regulations, and practice supports for medical office assistants in primary care: a scoping review
Source: BMC Prim Care. 2026 Feb 20;27:106. doi: 10.1186/s12875-026-03222-8 (PMC13032387; doi:10.1186/s12875-026-03222-8)
Supplement: Supplementary file 1 — Supplementary Material 1. [file 12875_2026_3222_MOESM1_ESM.docx]

**Appendix 1: Search Strategy - Medline**

Database(s): **Ovid MEDLINE(R) ALL** 1946 to July 11, 2023
Search Strategy:

| **#** | **Searches** | **Results** |
| --- | --- | --- |
| 1 | Primary Health Care/ | 91537 |
| 2 | Family practice/ or Family physicians/ | 80819 |
| 3 | (Primary care or General practi* or Primary health* or Family practice or Family medicine or primary medical care).tw,kf,jw. | 307488 |
| 4 | (general physician* or family physician* or family doctor* or family practitioner*).tw,kf. | 25052 |
| 5 | (family adj2 (nurse* or nursing)).tw,kf. | 3416 |
| 6 | (family adj2 (internist* or pediatrician* or paediatrician*)).tw,kf. | 1339 |
| 7 | or/1-6 [primary care] | 363311 |
| 8 | medical office assistant*.tw,kf. | 20 |
| 9 | medical assistant*.tw,kf. | 1164 |
| 10 | (receptionist* or medical reception or reception staff*).kf,tw. | 607 |
| 11 | office staff*.tw,kf. | 914 |
| 12 | office assistant*.tw,kf. | 57 |
| 13 | (front desk staff* or front line staff*).tw,kf. | 448 |
| 14 | ((office or offices) adj3 (secretar* or staff* or clerk or clerks)).kf,tw. | 1415 |
| 15 | ((administration* or administrative) adj3 (secretar* or staff* or worker* or clerk or clerks)).tw,kf. | 3071 |
| 16 | nonclinical staff*.tw,kf. | 122 |
| 17 | nonmedical staff*.tw,kf. | 82 |
| 18 | office administrat*.tw,kf. | 71 |
| 19 | clerical staff*.tw,kf. | 229 |
| 20 | administrat* assistant*.tw,kf. | 106 |
| 21 | Medical receptionist/ or Medical Secretaries/ | 491 |
| 22 | Practice Management/og | 385 |
| 23 | Allied Health Personnel/og | 307 |
| 24 | Health Personnel/og | 1722 |
| 25 | "Personnel Staffing and Scheduling"/og [Organization & Administration] | 3610 |
| 26 | or/8-25 [MOA] | 13461 |
| 27 | 7 and 26 | 1980 |
| 28 | (((primary care or primary healthcare or primary health care or general practice or family practice) adj3 (personnel* or interdisciplinary team* or multidisciplinary team*)) and (clinic or clinics or office or offices)).tw. | 70 |
| 29 | 27 or 28 | 2042 |
| 30 | (200* or 201* or 202*).dt,ez,da. | 23632828 |
| 31 | 29 and 30 | 1693 |

**Appendix 2: Articles not retrieved by Scopus**

| **Author** | **Title** |
| --- | --- |
| Jopson (2019) | Use of Apprenticeship to Meet Demand for Medical Assistants in the U.S. |
| Eden (2016) | EMPLOYED PRACTICE. Maximizing Your Medical Assistant's Role |

**Appendix 3a: Grey Literature Search Methods**

*Targeted Website Browsing/Searching*

|  | **Targeted Web Searches (Databases) in Google** | | |
| --- | --- | --- | --- |
|  |  | **Number of Results** | **Imported for study selection** |
| **Google.ca, Nov. 14^th^ 2023** | 1. medical office assistant AND primary care AND policy | **273,000,000** | **46** |
| **Google.ca, Nov. 14^th^ 2023** | 1. medical office assistant AND primary care AND regulation | **495,000,000** | **20** |
| **Google.ca, Nov. 14^th^ 2023** | 1. medical office assistant AND primary care AND guidelines | **215,000,000** | **10** |
| **Google.ca, Nov. 14^th^ 2023** | 1. medical office assistant AND primary care AND framework | **207,000,000** | **11** |
| **Google.ca, Nov. 22nd 2023** | 1. medical office assistant and primary care and tools | **394,000,000** | **26** |
| **Google.ca, Mar. 27^th^ 2024** | 1. medical office assistant AND primary care AND quintuple aim | **4,070,000** | **4** |
| **Google.ca, Mar. 27^th^ 2024** | 1. Administrative assistant AND primary care AND quintuple aim | **7,550,000** | **2** |
| **Google.ca, Mar. 27^th^ 2024** | 1. medical office assistant AND primary care AND quadruple aim | **391,000** | **0** |
| **Google.ca, Mar. 27^th^ 2024** | 1. Administrative assistant AND primary care AND quadruple aim | **537,000** | **0** |
| **Google.ca, Mar. 27^th^ 2024** | 1. secretary AND primary care AND policy | **97,500,000** | **12** |
| **Google.ca, Mar. 27^th^ 2024** | 1. receptionist AND primary care AND policy | **61,700,000** | **4** |

**Appendix 3b**

*Website Searching Documentation*

| **Date** | **Organization name & website URL** | **Search strategy(s)/ words searched including (if applicable) how items were selected*.*** | **# items retrieved/ search results** | **# of items screened (uploaded to citation management software)** |
| --- | --- | --- | --- | --- |
| Interventions, Policies, Statistics, Scope of Work etc. | | | | |
| Dec. 19^th^ 2023  (1.ab and 2.ab)  Oct 7^th^ 2024  (1.cde & 2.cde) | Women’s College Hospital | 1. Advanced Google 2. "medical office assistant" site:womenscollegehospital.ca 3. "medical assistant" site:womenscollegehospital.ca 4. “administrative assistant” site:womenscollegehospital.ca 5. “secretary” site:womenscollegehospital.ca 6. “receptionist” site:womenscollegehospital.ca 7. Site Searching 8. medical office assistant in womenscollegehospital.ca 9. medical assistant in womenscollegehospital.ca 10. administrative assistant in womenscollegehospital.ca 11. secretary in womenscollegehospital.ca 12. receptionist in womenscollegehospital.ca | 1.a) 203  b) 123  c) 13  d) 203  e) 4  2.a) 256  b) 219  c) 73  d) 23  e) 2  Total = 801 | 1.a) 0  b) 0  c) 0  d) 0  e) 0  2.a) 0  b) 0  c) 0  d) 0  e) 0 |
| Dec. 19^th^ 2023  (1.ab and 2.ab)  Oct 7^th^ 2024  (1.cde & 2.cde) | Ontario Health | 1. Advanced Google 2. "medical office assistant" site:ontariohealth.ca 3. "medical assistant" site:ontariohealth.ca 4. “administrative assistant” site:ontariohealth.ca 5. “secretary” site:ontariohealth.ca 6. “receptioinist” site:ontariohealth.ca 7. Site Searching 8. Medical office assistant in ontariohealth.ca 9. Medical assistant ontariohealth.ca 10. Administrative assistant ontariohealth.ca 11. Secretary ontariohealth.ca 12. Receptionist ontariohealth.ca | 1.a) 2  b) 131  c) 5  d) 49  e) 1  2.a) n/a  b) n/a  c) n/a  d) n/a  e) n/a  Total = 133 | 1.a) 1  b) 0  c) 0  d) 0  e) 0 |
| Dec. 19^th^ 2023  (1.ab and 2.ab)  Oct 7^th^ 2024  (1.cde & 2.cde) | Advanced Care Planning Canada | 1. Advanced Google 2. "medical office assistant" site:advancecareplanning.ca 3. "medical assistant" site:advancecareplanning.ca 4. “administrative assistant” site:advancecareplanning.ca 5. “Secretary” site:advancecareplanning.ca 6. “receptionist” site:advancecareplanning.ca 7. Site Searching 8. Medical office assistant in advancecareplanning.ca 9. Medical assistant in advancecareplanning.ca 10. Administrative assistant advancecareplanning.ca 11. Receptionist advancecareplanning.ca 12. Secretary advancecareplanning.ca | 1.a) 1  b) 49  c) 1  d) 0  e) 0  2.a) 1  2.b) 1  c) 1  d) 0  e) 0  Total = 52 | 1.a) 0  b) 0  c) 0  d) 0  e) 0  2.a) 0  b) 0  c) 0  d) 0  e) 0 |
| Dec. 19^th^ 2023  (1.ab and 2.ab)  Oct 7^th^ 2024  (1.cde & 2.cde) | Healthforce Centre at UCSF | 1. Site Searching 2. medical office assistant in healthforce.ucsf.edu 3. medical assistants in healthforce.ucsf.edu (NOT singular) 4. administrative assistant in healthforce.ucsf.edu 5. secretary in healthforce.ucsf.edu 6. receptionist in healthforce.ucsf.edu 7. Advanced Google 8. medical office assistant site:healthforce.ucsf.ed 9. "medical assistant" site:healthforce.ucsf.edu 10. “administrative assistant” site:healthforce.ucsf.edu 11. “secretary” site:healthforce.ucsf.edu 12. “receptionist” site:healthforce.ucsf.edu | 1.a) 6  1.b) 59  c) 5  d) 5  e) 0  2.a)0  b) 109  c) 10  d) 79  e) 10  Total = 174 | 1.a) 0  b) 22  c) 0  d) 0  e) 0  2.a) 0  b) 22 (duplicates)  c) 1 (duplicate)  d) 0  e) 0 |
| Dec. 19^th^ 2023  (1.ab and 2.ab)  Oct 7^th^ 2024  (1.cde & 2.cde) | Ontario Medical Association | 1. Advanced Google 2. "medical office assistant" site:oma.org 3. medical assistant site:oma.org 4. “administrative assistant” site:oma.org 5. “secretary” site:oma.org 6. “receptionist” site:oma.org 7. Site Searching 8. Medical office assistant in oma.org 9. Medical assistant in oma.org 10. Administrative assistant in oma.org 11. Secretary in oma.org 12. Receptionist in oma.org | 1.a) 76  b) 202  c) 4  d) 79  e) 77  2.a) 1056  b) 1040  c) 505  d) 58  e) 13  Total = 2374 | 1.a) 0  b) 0  c) 0  d) 0  e) 0  2.a) 0  b) 0  c) 0  d) 0  e) 0 |
| Dec. 19^th^ 2023  (1.ab and 2.ab)  Oct 7^th^ 2024  (1.cde & 2.cde) | Divisions of Family Practice | 1. Advanced Google 2. "medical office assistant" site:divisionsbc.ca 3. "medical assistant" site:divisionsbc.ca 4. “administrative assistant” site:divisionsbc.ca 5. “secretary” site:divisionsbc.ca 6. “receptionist” site:divisionsbc.ca 7. Site Searching 8. Medical office assistant in divisionsbc.ca 9. Medical assistant in divisionsbc.ca 10. Administrative assistant in divisionsbc.ca 11. Secretary in divisionsbc.ca 12. Receptionist in divisionsbc.ca | 1.a) 385  b) 5  c) 402  d) 476  e) 33  2.a) 40  b) 62  c) 59  d) 46  e) 2  Total = 492 | 1.a) 1  b) 0  c) 0  d) 0  e) 0  2.a) 0  2.b) 0  c) 0  d) 0  e) 0 |
| Dec. 19^th^ 2023  (1.ab and 2.ab)  Oct 7^th^ 2024  (1.cde & 2.cde) | Agency for Health Care Research and Quality | 1. Advanced Google 2. “medical office assistant” site:ahrq.gov 3. “medical assistant” site:ahrq.gov 4. “administrative assistant” site:ahrq.gov 5. “secretary” site:ahrq.gov 6. “receptionist” site:ahrq.gov 7. Site Searching 8. medical office assistant in ahrq.gov 9. medical assistant in ahrq.gov 10. administrative assistant in ahrq.gov 11. secretary in ahrq.gov 12. receptionist in ahrq.gov | 1.a) 1  b) 422  c) 125  d) 7330  e) 466  2.a) 10,000  2.b) 10,000  c) 10,000  d) 4048  e) 1368 | 1.a) 0  b) 1  c) 0  d) 0  e) 0  2.a) 0  b) 0 (1 duplicate)  c) 0  d) 0  e) 0 |
| Dec. 19^th^ 2023  (1.ab and 2.ab)  Oct 7^th^ 2024  (1.cde & 2.cde) | UW Department of Family Medicine | 1. Advanced Google 2. Medical Office Assistant ("medical office assistant" site:familymedicine.uw.edu) 3. Medical Assistant ("medical assistant" site:familymedicine.uw.edu) 4. "Administrative assistant " site:familymedicine.uw.edu 5. "secretary" site:familymedicine.uw.edu 6. "receptionist" site:familymedicine.uw.edu 7. Site Searching 8. Medical Office Assistant in familymedicine.uw.edu 9. Medical Assistant in familymedicine.uw.edu 10. Administrative assistant in familymedicine.uw.edu 11. Secretary in familymedicine.uw.edu 12. Receptionist in familymedicine.uw.edu | 1.a) 1  b) 162  c) 7  d) 45  e) 7  2.a) 10  2.b) 10  c) 10  d) 5  e) 2  Total = 183 | 1.a) 0  b) 6  c) 0  d) 0  e) 0  2.a) 0  2.b) 0  c) 0  d) 0  e) 0 |
| Dec. 19^th^ 2023  (1.ab and 2.ab)  Oct 7^th^ 2024  (1.cde & 2.cde) | Family Practices Services Committee (FPSC) | 1. Advanced Google 2. "medical office assistant" site:fpscbc.ca 3. "medical assistant" site:fpscbc.ca 4. "Administrative assistant " site:fpscbc.ca 5. "secretary" site:fpscbc.ca 6. "receptionist" site:fpscbc.ca 7. Site Search 8. Medical Office Assistant in fpscb.ca 9. Medical Assistant in fpscb.ca 10. Administrative assistant in fpscb.ca 11. Secretary in fpscb.ca 12. Receptionist in fpscb.ca | 1.a) 48  1.b) 71  c) 0  d) 1  e) 0  2. a) 13  2.b) 20  c) 2  d) 1  e) 0  Total = 152 | 1.a) 1  1. b) 0 (1 duplicate)  c) 0  d) 0  e) 0  2.a) 0  2.b) 0  c) 0  d) 0  e) 0 |
| Dec. 19^th^ 2023  (1.ab and 2.ab)  Oct 7^th^ 2024  (1.cde & 2.cde) | Vancouver Coastal Health | 1. Advanced Google 2. "medical office assistant" site:vch.ca 3. "medical assistant" site:vch.ca 4. "Administrative assistant " site:vch.ca 5. "secretary" site:vch.ca 6. "receptionist" site:vch.ca 7. Site Searching 8. Medical office assistant in vch.ca 9. Medical assistant in vch.ca 10. Administrative assistant in vch.ca 11. Secretary in vch.ca 12. Receptionist in vch.ca | 1.a) 49  1.b) 1  c) 41  d) 50  e) 10  2.a) 53  2.b) 224  c) 11  d) 6  e) 2  Total = 327 | 1.a) 0  1.b) 0  c) 0  d) 0  e) 0  2.a) 0  2.b) 0  c) 0  d) 0  e) 0 |
| Dec. 19^th^ 2023  (1.ab and 2.ab)  Oct 7^th^ 2024  (1.cde & 2.cde) | US Bureau of Labor Statistics | 1. Advanced Google 2. "medical office assistant" site:bls.gov 3. "medical assistant" site:bls.gov 4. "Administrative assistant " site:bls.gov 5. "secretary" site:bls.gov 6. "receptionist" site:bls.gov 7. Site Searching 8. medical office assistant in bls.gov 9. medical assistant in bls.gov 10. administrative assistant in bls.gov 11. secretary in bls.gov 12. receptionist in bls.gov | 1.a) 5840  1.b) 79  c) 68  d) 1880  e) 212  2.a) 10,400  2.b) 14,400  c) 11,700  d) 1,700  e) 8,180  Total = 30,719 | 1.a) 3  1.b) 0 (2 duplicates)  c) 0  d) 0  e) 0  2.a) 0 (3 duplicates)  2.b) 0 (2 duplicates)  c) 0  d) 0  e) 0 |
| Dec. 19^th^ 2023  (1.ab and 2.ab)  Oct 7^th^ 2024  (1.cde & 2.cde) | Sage Journals | This will not be included because we have already done a database search for scholarly literature – the article retrieved from the Google search has already been uploaded and screened in Covidence. |  |  |
| Dec. 19^th^ 2023  (1.ab and 2.ab)  Oct 7^th^ 2024  (1.cde & 2.cde) | The Doctor’s Company | 1. Advanced Google 2. "medical office assistant" site:thedoctors.com 3. "medical assistant" site:thedoctors.com 4. "Administrative assistant " site:thedoctors.com 5. "secretary" site:thedoctors.com 6. "receptionist" site:thedoctors.com 7. Site Searching 8. Medical office assistant in thedoctors.com 9. Medical assistant in thedoctors.com 10. Administrative assistant in thedoctors.com 11. Secretary in thedoctors.com 12. Receptionist in thedoctors.com | 1.a) 118  1.b) 33  c) 37  d) 42  e) 6  2.a) 580  2.b) 550  c) 71  d) 2  e) 3  Total = 1281 | 1.a) 1  1.b) 0 (1 duplicate)  c) 0  d) 0  e) 0  2.a) 0 (1 duplicate)  2.b) 0 (1 duplicate)  c) 0  d) 0  e) 0 |
| Dec. 19^th^ 2023  (1.ab and 2.ab)  Oct 7^th^ 2024  (1.cde & 2.cde) | Government of Canada (job bank) | 1. Advanced Google 2. "medical office assistant" site:noc.esdc.gc.ca 3. "medical assistant" site:noc.esdc.gc.ca 4. "Administrative assistant " site:noc.esdc.gc.ca 5. "secretary " site:noc.esdc.gc.ca 6. "receptionist" site:noc.esdc.gc.ca 7. Site Searching 8. Medical office assistant in noc.esdc.gc.ca 9. Medical assistant in noc.esdc.gc.ca 10. Administrative assistant in noc.esdc.gc.ca 11. Administrative assistant in noc.esdc.gc.ca 12. Secretary in noc.esdc.gc.ca 13. Receptionist in noc.esdc.gc.ca | 1.a) 1  1.b) 46  c) 52  d) 270  e) 76  2.a) 1  2.b) 0  c) 66,446  d) 116,474  e) 718  Total = 48 | 1.a) 1  1.b) 0  c) 0  d) 0  e) 0  2.a) 0 (1 duplicate)  2.b) 0  c) 0  d) 0  e) 0 |
| Dec. 19^th^ 2023  (1.ab and 2.ab)  Oct 7^th^ 2024  (1.cde & 2.cde) | CFP | 1. Advanced Google 2. "medical office assistant" site:cfp.ca 3. "medical assistant" site:cfp.ca 4. "Administrative assistant " site:noc.esdc.gc.ca 5. "secretary" site:noc.esdc.gc.ca 6. "receptionist" site:noc.esdc.gc.ca 7. Site Searching 8. Medical office assistant in cfp.ca 9. Medical assistant in cfp.ca 10. Administrative assistant in cfp.ca 11. Secretary in cfp.ca 12. Receptionist in cfp.ca | 1.a) 32  1.b) 7  c) 52  d) 306  e) 76  2.a) 22  2.b) 79  c) 704  d) 0  e) 62  Total = 140 | 1.a) 0 (1 duplicate)  1.b) 0  c) 0  d) 0  e) 0  2.a) 1 (2 duplicates)  2.b) 0  c) 0  d) 0  e) 0 |
| Dec. 19^th^ 2023  (1.ab and 2.ab)  Oct 7^th^ 2024  (1.cde & 2.cde) | Medical Board of California | 1. Advanced Google 2. "medical office assistant" site:mbc.ca.gov 3. "medical assistant" site:mbc.ca.gov 4. "Administrative assistant " site:mbc.ca.gov 5. "secretary" site:mbc.ca.gov 6. "receptionist" site:mbc.ca.gov 7. Site Searching 8. Medical office assistant in mbc.ca.gov 9. Medical assistant in mbc.ca.gov 10. Administrative assistant in mbc.ca.gov 11. Secretary in mbc.ca.gov 12. Receptionist in mbc.ca.gov | 1.a) 3040  1.b)  c) 181  d) 1080  e) 70  2.a) 5120  2.b) 4970  c)  3,570  d) 982  e) 65  Total = 13,702 | 1.a) 3  1.b) 0  2.a) 0 (1 duplicate)  2.b) 0 (2 duplicates)  c) 0 (1 duplicate)  d) 0  e) 0 (1 duplicate) |
|  |  |  | 70,578 | 41 included articles added to covidence, 41 duplicates, 70537 articles removed |

**Appendix 3c**

*Grey Literature Database Search*

| **Date** | **Database name & URL** | **Search strategy(s)/ words searched including (if applicable) how items were selected*.*** | **# of items retrieved/ search results** | **# of items screened (uploaded to citation management software)** |
| --- | --- | --- | --- | --- |
| Nov. 24^th^ 2023 | Proquest Dissertations/ Theses Global  [Advanced Search - ProQuest](https://www.proquest.com/advanced?accountid=37543) | (noft("general physician*" or "family physician*" or "family doctor*" or "family practitioner*") OR noft("Primary care" or "General practi*" or "Primary health*" or "Family practice*" or "Family medicine*" or "primary medical care") OR noft(family NEAR/2 (nurse* or nursing)) OR noft(family NEAR/2 (internist* or pediatrician* or paediatrician*))) **AND** (noft("medical office assistant*" ) OR noft("medical assistant*" ) OR noft(receptionist* or "medical reception" or "reception staff*") OR noft("office staff*") OR noft("office assistant*") OR noft("front line staff*") OR noft((office or offices) NEAR/3 (secretar* or staff* or clerk or clerks)) OR noft((administration* or administrative) NEAR/3 (secretar* or staff* or worker* or clerk or clerks)) OR noft("nonclinical staff*" OR "nonmedical staff*"  OR "office administrat*" OR "clerical staff*" OR "administrat* assistant*" ))  **OR**  (("primary care*" or "primary healthcare*" or "primary health care*" or "general practice*" or "family practice*") NEAR/3 (personnel* or "interdisciplinary team*" or "multidisciplinary team*")) and (clinic or clinics or office or offices) | 1297 | 152 |
| Oct. 18^th^, 2023 | Open Grey | 1. "medical office assistant AND "primary care" 2. "office assistant" and "primary care" 3. "primary care office" and staff 4. primary care office AND regulations 5. primary care office AND policies 6. medical receptionist AND regulations 7. medical receptionist AND policies 8. medical office assistant AND regulations 9. medical office assistant AND policies | 0 results for all search strings | 0 |
| Oct. 18^th^, 2023 | New York Academy of Medicine Grey Literature Report | Medical office assistant OR medical secretary | 115 results | 0 |

**Appendix 3d**

*Search Engine Searching*

| **Date** | **Search engine** | **Search strategy(s) including how items were selected** | **# of results per search** | **Duplicates** | **# of items screened (uploaded to citation management software)** |
| --- | --- | --- | --- | --- | --- |
| Nov. 29th 2023 | Google Scholar | 1. "medical office assistant" AND "primary care" policy OR regulation OR guidelines OR frameworks OR tools | 279 | 15 > till page 10 | 11 |
| Dec. 5^th^ 2023 |  | 1. regulations OR policy OR guidelines OR frameworks OR tools "receptionist" "primary care" | 13,300 | 30 > till page 10 | 11 |
| Dec. 6^th^ 2023 |  | 1. "administrative support staff" AND "primary care" policy OR regulation OR guidelines OR frameworks OR tools | 1060 | 15 > till page 10 | 21 |
|  |  |  | 14639 | 60 | 43 included, 14596 excluded |

**Appendix 3e**

*Duckduckgo Search Strings*

| Date Searched | MOA Terms | Primary Care Terms | Supports Terms | Full Search String | How many new relevant articles | How many duplicates | Items included to screen |
| --- | --- | --- | --- | --- | --- | --- | --- |
| Nov. 4 2024 | Medical Office assistant | X | Policy | ~"medical office assistant" policy | 0 | 2 | 0 |
| Nov. 4 2024 | Medical office assistant | X | policy OR regulation OR guideline OR framework OR tool | ~"medical office assistant" policy OR regulation OR guideline OR framework OR tool | 1 | 0 | 0 |
| Nov. 4 2024 | Administrative Assistant | Primary Care Setting | Policy | ~"administrative assistant" policy in primary care setting | 10 | 7 | 0 |
| Nov. 7 2024 | Medical Office Assistant | Primary Care Setting | Policy | ~"medical office assistant " policy in primary care setting | 1 | 15 | 0 |
| Nov. 7 2024 | Secretary | Primary Care Setting | Policy | ~"Secretary " policy in primary care setting | 7 | 2 | 0 |
| Nov. 7 2024 | Administration | Primary Care Setting | Policy | ~"administration " policy in primary care setting | 5 | 5 | 1 |
| Nov. 7 2024 | Receptionist | Primary Care Setting | Policy | ~"Receptionist " policy in primary care setting | 9 | 18 | 3 |
| Nov. 11 2024 | Receptionist | Primary Care Setting | Regulation | ~"receptionist " regulation in primary care setting | 0 | 19 | 0 |
| Nov. 11 2024 | Secretary | Primary Care Setting | Regulation | ~"secretary " regulation in primary care setting | 0 | 7 | 0 |
| Nov. 11 2024 | Medical Office Assistant | Primary Care Setting | Regulation | ~"medical office assistant " regulation in primary care setting | 1 | 15 | 1 |
| Nov. 11 2024 | Administrative Assistant | Primary Care Setting | Regulation | ~"administrative assistant " regulation in primary care setting | 0 | 9 | 0 |
| Nov. 13 2024 | Administrative Personnel | Primary Care Setting | Policy | ~"administrative personnel " policy in primary care setting | 12 | 10 | 2 |
| Nov. 20 2024 | Medical Office Staff | Primary Care Setting | Policy | ~"medical office staff " policy in primary care setting | 16 | 8 | 5 |
| Nov. 21 2024 | Office Administrator | Primary Care Setting | Policy | ~"office administrator " policy in primary care setting | 9 | 1 | 1 |
| Nov 21 2024 | Medical Assistant | Primary Care Setting | Policy | ~"medical assistant " policy in primary care setting | 2 | 16 | 1 |
| Nov. 25 2024 | Medical Administrator | Primary Care Setting | Policy | ~" Medical Administrator " policy in primary care setting | 6 | 9 | 0 |
| Nov. 25 2024 | Administrative Personnel | Primary Care Setting | guideline | ~"Administrative Personnel " guideline in primary care setting | 0 | 11 | 0 |
| Nov. 25 2024 | Administrative Personnel | Primary Care Setting | framework | ~"Administrative Personnel " framework in primary care setting | 0 | 11 | 0 |
| Nov. 25 2024 | Medical Office Staff | Primary Care Setting | guideline | ~" Medical Office Staff " guideline in primary care setting | 0 | 14 | 0 |
| Nov. 25 2024 | Medical Office Staff | Primary Care Setting | framework | ~" Medical Office Staff " framework in primary care setting | 3 | 1 | 3 |
| Nov. 25 2024 | Office Administrator | Primary Care Setting | guideline | ~" Office Administrator " guideline in primary care setting | 0 | 8 | 0 |
| Nov. 25 2024 | Office Administrator | Primary Care Setting | framework | ~" Office Administrator " framework in primary care setting | 0 | 13 | 0 |
| Nov. 25 2024 | Medical Assistant | Primary Care Setting | guideline | ~"Medical Assistant" guideline in primary care setting | 0 | 11 | 0 |
| Nov. 25 2024 | Medical Assistant | Primary Care Setting | framework | ~"Medical Assistant" framework in primary care setting | 0 | 16 | 0 |
| Nov. 25 2024 | Medical Administrator | Primary Care Setting | guideline | ~" Medical Administrator " guideline in primary care setting | 1 | 4 | 1 |
| Nov. 25 2024 | Medical Administrator | Primary Care Setting | framework | ~" Medical Administrator " framework in primary care setting | 0 | 9 | 0 |
| Nov. 28 2024 | Administrative Personnel | Family Medicine | Policy | ~" administrative personnel " policy in family medicine | 0 | 2 | 0 |
| Nov. 28 2024 | Medical Office Staff | Family Medicine | Policy | ~" Medical office staff" policy in family medicine | 0 | 1 | 0 |
| Nov. 28 2024 | Office Administrator | Family Medicine | Policy | ~" Office Administrator " policy in family medicine | 0 | 0 | 0 |
| Nov. 29 2024 | Medical Assistant | Family Medicine | Policy | ~" Medical Assistant " policy in family medicine | 1 | 3 | 0 |
| Nov. 29 2024 | Medical Administrator | Family Medicine | Policy | ~" Medical Administrator " policy in family medicine | 0 | 7 | 0 |
| Nov. 29 2024 | Administrative Personnel | Family Medicine | guideline/framework | ~" Administrative personnel " guideline or framework in family medicine | 0 | 5 | 0 |
| Nov. 29 2024 | Medical Office Staff | Family Medicine | guideline/framework | ~" Medical office staff" guideline or framework in family medicine | 1 | 12 | 1 |
| Nov. 29 2024 | Office Administrator | Family Medicine | guideline/framework | ~" office Administrator " guideline or framework in family medicine | 0 | 5 | 0 |
| Nov. 29 2024 | Medical Assistant | Family Medicine | guideline/framework | ~" Medical assistant " guideline or framework in family medicine | 0 | 9 | 0 |
| Nov. 29 2024 | Medical Administrator | Family Medicine | guideline/framework | ~" Medical Administrator " guideline or framework in family medicine | 0 | 5 | 0 |
| Nov. 29  2024 | Administrative Personnel | General Practice | Policy | ~" administrative personnel " policy in general care setting | 0 | 8 | 0 |
| Dec 2  2024 | Medical Office Staff | General Practice | Policy | ~" Medical office staff " policy in general practice setting | 0 | 4 | 0 |
| Dec 2  2024 | Office Administrator | General Practice | Policy | ~" Office Administrator " policy in general practice setting | 0 | 0 | 0 |
| Dec 2  2024 | Medical Assistant | General Practice | Policy | ~" Medical assistant " policy in general practice | 0 | 5 | 0 |
| Dec 2  2024 | Medical Administrator | General Practice | Policy | ~" Medical Administrator " policy in general practice | 0 | 0 | 0 |
| Dec 2  2024 | Administrative Personnel | General Practice | guideline/framework | ~" Administrative personnel" guideline or framework in general practice | 0 | 0 | 0 |
| Dec 2  2024 | Medical Office Staff | General Practice | guideline/framework | ~" Medical office staff " guideline or framework in general practice | 0 | 0 | 0 |
| Dec 2  2024 | Office Administrator | General Practice | guideline/framework | ~" office Administrator " guideline or framework in general practice | 0 | 0 | 0 |
| Dec 3 2024 | Medical Assistant | General Practice | guideline/framework | ~" Medical Assistant " guideline or framework in general practice | 0 | 2 | 0 |
| Dec 3 2024 | Medical Administrator | General Practice | guideline/framework | ~" Medical Administrator " guideline or framework in general practice | 0 | 4 | 0 |

**Appendix 4: Preliminary Search words for MOA program search**

| **OECD Country** | **MOA Job Terms Used** |
| --- | --- |
| Australia | Medical office assistant, receptionist |
| Austria | receptionist, medical administrative assistants |
| Belgium | Receptionist, medical secretary |
| Canada | Medical office assistant, medical administrative assistant, clinic receptionist, medical receptionist, medical office administrator |
| Chile | Medical assistant, secretary |
| Colombia | Healthcare administrator, medical assistant |
| Costa Rica | Medical assistant, medical office manager |
| Czech Republic | Administrative assistant, medical assistant |
| Denmark | Clinic secretary, medical secretary |
| Estonia | Receptionist, administrator |
| Finland | healthcare reception clerk, reception clerk, receptionist |
| France | Medical secretary, administrative assistant |
| Germany | “Medizinische Fachangestellte (MFAs),” medical assistant, doctor’s assistant |
| Greece | Medical office secretary, clinic secretary, office admin |
| Hungary | Receptionist, healthcare secretary, office admin |
| Iceland | Receptionist, medical secretary |
| Ireland | Receptionist, medical secretary |
| Israel | Receptionist, medical secretary |
| Italy | Receptionist, medical secretary |
| Japan | medical office administrator, receptionist |
| Korea | medical office administrator, receptionist, registration clerk |
| Latvia | Receptionist, medical secretary |
| Lithuania | Receptionist, medical secretary |
| Luxembourg | Receptionist, medical secretary |
| Mexico | Receptionist, medical secretary |
| Netherlands | Receptionist, medical secretary |
| New Zealand | Medical office assistant, medical receptionist, medical administrator |
| Norway | Medical office assistant, doctor’s office assistant, receptionist |
| Poland | Medical receptionist, receptionist |
| Portugal | Receptionist, medical secretary |
| Slovak Republic | Receptionist, administrator |
| Slovenia | Receptionist, medical secretary |
| Spain | Receptionist, medical secretary |
| Sweden | Receptionist, medical secretary |
| Switzerland | Medical practice assistant, receptionist, medical secretary |
| Türkiye | Receptionist, medical secretary |
| United Kingdom | Medical receptionist, medical secretary, healthcare administrator |
| USA | Medical assistant, medical secretary, receptionist |

**Appendix 5: Description of MOA Program in OECD countries**

| OECD COUNTRY | # of MOA Programs Reviewed | Skills & Training | | | | | | Program Components | | |
| --- | --- | --- | --- | --- | --- | --- | --- | --- | --- | --- |
|  |  | **Communication** | **EMR** | **Romming/** **Clinical** | **Ethics/** **Privacy** | **Administrative** | **First Aid Training** | **Has a Practicum Component** | | **Mode of Learning (AS/in-person)** |
| Australia | 5 | 5 | 3 | 3 | 5 | 5 | 1 | 1 | AS (3) Hybrid (1)  In-person (1) | |
| Austria | 3 | 2 | 1 | 1 | 3 | 2 | 1 | 2 | In-person (3) | |
| Belgium | 4 | 3 | 1 | 2 | 2 | 4 | 1 | 1 | AS (2)  In-person (2) | |
| Canada | 5 | 5 | 5 | 5 | 5 | 5 | 3 | 4 | AS (2) In-person (3) | |
| Colombia | 3 | 2 | 0 | 2 | 2 | 1 | 0 | 0 | In-person (3) | |
| Costa Rica | 1 | 0 | 1 | 1 | 1 | 0 | 0 | 1 | In-person (1) | |
| Denmark | 2 | 2 | 0 | 1 | 2 | 2 | 1 | 1 | AS (1)  In-person (1) | |
| France | 3 | 3 | 1 | 0 | 1 | 3 | 0 | 0 | AS (1) In-person (2) | |
| Germany | 4 | 3 | 2 | 3 | 1 | 3 | 1 | 3 | In-person (4) | |
| Ireland | 4 | 4 | 2 | 1 | 4 | 4 | 1 | 1 | AS (3) In-person (1) | |
| Israel | 1 | 0 | 0 | 0 | 0 | 0 | 1 | 0 | AS (1) | |
| Japan | 2 | 2 | 2 | 2 | 2 | 2 | 1 | 1 | In-person (2) | |
| Lithuania | 1 | 0 | 0 | 0 | 0 | 0 | 0 | 0 | In-person (1) | |
| Mexico | 1 | 1 | 1 | 1 | 0 | 1 | 0 | 1 | In-person (1) | |
| Netherlands | 1 | 1 | 0 | 1 | 0 | 1 | 1 | 1 | AS (1) | |
| New Zealand | 4 | 4 | 1 | 1 | 2 | 3 | 1 | 0 | AS (4) | |
| Norway | 2 | 1 | 1 | 1 | 1 | 1 | 1 | 1 | In-person (2) | |
| Portugal | 1 | 1 | 0 | 1 | 1 | 1 | 1 | 0 | AS (1) | |
| Switzerland | 2 | 2 | 1 | 1 | 2 | 2 | 0 | 0 | In-person (2) | |
| Turkey | 4 | 3 | 0 | 1 | 3 | 3 | 0 | 1 | In-person (4) | |
| United Kingdom | 5 | 5 | 0 | 0 | 4 | 5 | 0 | 0 | AS (4)  Hybrid (1) | |
| United States | 5 | 5 | 3 | 0 | 5 | 5 | 0 | 0 | AS (3) In-person (2) | |

AS=asynchronous

*No programs were found in Chile, Czech Republic, Estonia, Finland, Greece, Hungary, Iceland, Italy, Korea, Latvia, Luxembourg, Poland, Slovak Republic, Slovenia, Spain, and Sweden

**Appendix 6. Key Findings Tables**

| **GREEN** | **Positive outcome** |
| --- | --- |
| **PINK** | **No significant change** |
| **YELLOW** | **Negative outcome** |

**Table A1 Interventions: Education, Counselling, Health Coaching**

|  |  | Impact on the Quintuple Aim | | | | |
| --- | --- | --- | --- | --- | --- | --- |
| Author (Year) | Intervention details | Patient Care | Workforce Well-Being | Population Health | Cost of Care/ Efficiency | Equity |
| AuYoung (2015)  **USA** | Primary care practice was redesigned to **improve patient self-management of obesity as MAs take on new roles of health coaches.** | Positive patient response to extended MOA roles | Support from Clinic Staff |  |  |  |
| Bass (2013)  **Canada** | Clinical preventative care using a Tobacco Intervention Flow Sheet: Front-office staff **to provide tobacco interventions (e.g., screening, chart reminders, self-management plans)**. | Patient enthusiasm regarding new role  Increased documentation of patients at risk | Clinician enthusiasm regarding new role |  |  |  |
| Clark (2022)  **USA** | **MA coaches identified patients for health coaching and then received health coaching and self-management support.** | Patient perceptions of chronic illness | MA confidence in delivering intervention |  |  | Younger, Hispanic, Spanish speaking, unemployed, lower SES patients had an average of 3 more follow-ups than the other group |
| Celeste-Harris (2006)  **USA** | Trained MOAs to assist with diabetic care (e.g., talked **with patients about basic medical aspects of diabetes, self-monitoring** of blood glucose, meal planning, and exercise). | Patient satisfaction; Patient confidence in MAs | Provider satisfaction; Office staff satisfaction | A1C ; blood pressure; Diabetics evaluation Patient quality of life; Patient optimism |  |  |
| Djuric (2017)  USA | MAs trained as health coaches to support patients with **sleep, diet, physical activity** and **behaviour change goals, and took measurements** (e.g., blood pressure, weight and height). | Patient satisfaction with care | MA satisfaction; Provider Satisfaction | Body mass index; Waist circumference; blood pressure; Confidence to carry out physical activity; Sleep quality; Total physical activity |  |  |
| Dunn (2023)  USA | Implementation of electronic reminders for depression screening and suicide assessment and providing electronic consultation access to a psychiatrist to ease front desk workload | Behavioural health screening | staff confidence in identifying behavioural health needs; Staff confidence in providing good care for behavioral health concern; Staff confidence addressing needs for patients with chronic conditions |  |  |  |
| Rodriguez (2018)  USA | MA with expanded roles and responsibilities for managing diabetes care: **providing patient education, goal setting, action planning, and routine monitoring.** | Improved chronic care experiences among patients treated in the MA led intervention arm compared to control group |  | MA Intervention: LDL-C ; MA Intervention: HbA1c testing; Control group patients reported fewer symptoms of diabetes-related emotional distress |  |  |
| Maryniuk (2013)  USA | Medical office staff to assist with diabetic care: **talk with patients about basic medical aspects of diabetes, self-monitoring of blood glucose, meal planning, and exercise and provided community and support resources.** | Increased frequency MOS talked with patients; Improvements in number of office system activities conducted for diabetes care, including use of flow sheets and provision of educational handouts | Physician satisfaction with the program | A1C; Blood pressure; LDL cholesterol; Microalbumin rate; Glomerular filtration rate |  |  |
| Naughton (2013) USA | Increased MA to MD (medical doctor) ratio, MAs were trained for routine tests and preventative care |  |  |  |  |  |
| Nguyen (2016), USA | MA were trained on how to indicate a patient’s current and target blood pressure. |  |  | Mean systolic blood pressure; Mean diastolic blood pressure; Rate of intervention completion; completed |  |  |
| Ricketts (2016), United Kingdom | Implemented a Chlamydia support worker, training workshop for staff, supporting resources and materials, bimonthly newsletters, a team champion to increase chlamydia screening in under 25-year-olds | Patient responses to MAs | Awareness of health topic and confidence in providing care |  |  |  |
| White (2008), United Kingdom | Educational workshop to educate MAs on mental health, raise awareness of patient depression at the front desk, examined potential barriers and ways of improving their access to primary healthcare. |  | Knowledge of depression; |  |  |  |
| Lieu (2022), USA | Encouraged video visits as the initial mode of interaction between staff and patients. MAs **developed new approaches to empathize, tailor interactions with patients, and overcome barriers to connecting via video.** |  |  |  |  |  |
| Lisel B. (2015), USA | New roles for MAs to capitalize on their language capacity to provide health coaching and health navigation services for diabetic patients |  | MA Career impacts | Patients with low HbA1C levels receive health coaching; Hypertension control | Organizational efficiency | Increased opportunities for bilingual MAs; supports patients who need culturally appropriate health services |
| Munro (2019)  **USA** | Clinic staff trained in shared decision making about contraceptive methods: **use decision aids + training, or a video + prompt card.** |  | MA Communication Skills; |  |  |  |

**Table A2 Navigator or Care Management of Patients (8 articles)**

|  |  | Impact on the Quintuple Aim | | | | |
| --- | --- | --- | --- | --- | --- | --- |
| Author (Year) | Intervention details | Patient/Care Experience | Workforce Well-Being | Population Health | Cost of Care/ Efficiency | Equity |
| Ansorg (2022)  **Switzerland** | Created clinical specialized medical assistants, trained **to care for chronic conditions like diabetes** | Patient satisfaction; Treatment Burden | Task shifting and better functioning; Positive feedback | SGED scores (a diabetes management measurement tool) |  |  |
| Black (2013)  **Australia** | Non-GP staff in chronic disease management help improve patient care by **organizing appointments and managing recall systems for conditions like diabetes.** | patient-assessed quality of care | team profile (PPI) scores (by admin staff and nurses) |  |  |  |
| Brain (2017)  **USA** | Utilized MAs to **provide initial weight-loss counseling to patients**. Tasks include **identifying patients at risk, provide community resources, and referral to nurses for follow-up.** | patient satisfaction; Patient comfort; Correct referral to clinical nurse | Time constraint and staffing challenges | BMI scores; satisfaction with health care experiences | employee retention rate; Emergency department visits, urgent care vists, hospital admissions, hospital visits; Dropped calls at MA phone back |  |
| Gensichen (2016)  **Germany** | MAs delivered protocol-based care management (i.e., assessment, action planning, and monitoring) for patients at high risk for future hospitalization. **Trained in behavioral counseling strategies, symptom monitoring, medication assessment, and interprofessional communication.** |  |  | mean hospitalizations for patients with COPD diagnosis; quality of life physical component; quality of life mental component |  |  |
| Gensichen (2009)  **German**y | Integrated health care assistants (HCAs) into depression management in small primary care practices. **HCAs were trained in the basics of clinical depression and were taught communication skills using interactive methods** |  | MA Job satisfaction; MA Work confidence |  |  |  |
| Lyon (2018)  **USA** | Under the new team-based care model, increased the ratio of MAs to providers and expanded the role of MAs throughout the patient visit. | Staff and provider communication | Provider burnout; staff experiences | Increased screening for colon cancer; breast cancer, hypertension control and other diabetes clinical measures;  significant improvement in clinical quality measures | Increased visit volume, Provider overtime hours decreased |  |
| MacKay (2014)  **Canada** | MOAs saw patients in 20-minute appointments for **measurement of height, weight, (BMI), waist circumference and blood pressure; a screening diabetic foot examination; and ordered routine laboratory tests for diabetic care** | Patient satisfaction surveys,  access improved,  staff and provider communication improved | Increased willingness to participate and initiate quality improvement activities; Improved satisfaction; Efficiency;  Team functioning ;  Inbox management from providers; |  | Visit volume covered additional MA staff cost |  |
| Lisel B. (2011) **USA** | MAs trained to **provide risk assessments and care coordination to elderly patient**s, and to conduct home visits to elderly patients in remote rural areas. |  | Lower MA turnover |  | Increased patient volume + decreased cost |  |
| Brunton (2022), United Kingdom | Medical office staff and managers are given care navigation (CN) and social prescribing link worker (SPLW) roles, to introduce ‘active signposting’, to direct patients to the right professional/services at the right time and free up GP time. |  |  |  |  |  |
| Siddiqui (2017),  United Kingdom | Evaluated the nature of telephone consultations undertaken by GPs at two practices and whether patients’ needs could have adequately been met without the involvement of a GP. |  |  |  |  |  |

**Table A3 Screening Activities (5 articles)**

|  |  | **Impact on the Quintuple Aim** | | | | |
| --- | --- | --- | --- | --- | --- | --- |
| Author (Year)  Country | Intervention details | Patient/Care Experience | Workforce Well-Being | Population Health | Cost of Care/ Efficiency | Equity |
| Ferrer (2009) **USA** | MA based program that directed screening & referrals of 4 health behaviors (smoking, drinking, diet, inactivity). |  | MA satisfaction | Patient screening | MA Referral Rates |  |
| Ishida (2019)  **USA** | Care Coordinator role created to increase CRC screening  using phone outreach and staff assistance. **MAs would identify who was due for screening, and to complete intake for those who needed to be scheduled.** | Patient's understanding of rationale behind the automated messaging | MA dissatisfaction to practice change | Increase in patients completing the advanced care planning discussions | Wait time + Time spent with staff |  |
| Lock (2000)  **United Kingdom** | Receptionists received training for screening and brief alcohol intervention, to hand out and **explain an ‘alcohol use disorders identification test’ to patients.** |  | Changes in receptionist attitudes during programme |  |  |  |
| Katz (2004)  **USA** | Smoking cessation intervention: MAs **providing real-time reminders, self-help materials, and proactive telephone counseling** | MA patients were significantly less likely than RN patients to be asked about willingness to quit | MA willingness to learn & take on new tasks;  Satisfaction with role |  |  |  |
| Gorman (2021)  **USA** | MAs responsible for completing depression screening test with patients and follow-up steps including additional questions + flagging their EHR for critical results. |  |  | MA screening rates in comparison to physician screening rates;  Differences by age and insurance type improved  to a lesser degree |  | MA depression screening amongst Black/African American adults  vs. White patients (in comparison to physician-only screening); visits by Asians vs. White patients (before and after the MA screening protocol); |
| Herrera (2019)  **USA** | MAs helped facilitate screening patients for social determinants of health during office visits. t and the referral process with primary care providers. | resources provided had limited patient utility | Helped staff practice holistic care; staff ability to connect patients to social services; intervention implementation into daily activities; MA confusion about intervention affected workflow |  |  | Offered in multiple language; Families offered social services info |

**Table A4 Advanced Rooming (2 articles)**

|  |  | **Impact on the Quintuple Aim** | | | | |
| --- | --- | --- | --- | --- | --- | --- |
| Author (Year)  Country | Intervention details | Patient/Care Experience | Workforce Well-Being | Population Health | Cost of Care/ Efficiency | Equity |
| Gray (2016)  **USA** | Flow managers: MAs facilitated clinic efficiency by guiding their physician through the day and taking on administrative tasks that previously fell solely on physicians. |  | MA dissatisfaction with lack of financial reward for increased contribution; MA skills development;  Power dynamics with MDs & MAs |  |  |  |
| Harper (2023)  **USA** | MAs addressed **preventive services, obtained a preliminary visit agenda, and completed a warm hand-off to the provider**. In Visit Assistance, MAs stayed in the room the entire visit to **assist with the visit workflow.** | Patients satisfied with visit assistance;  Patients were satisfied with Enhanced Rooming | MA confidence;  physician confidence and satisfaction | Improved mammogram ordering rates;  Visit turnaround time shorter (more smooth clinic flow) |  |  |
| Cuenca (2018),  USA | Implementing team rooming where a physician and MA reverse certain roles during a patient visit. MA conducts the initial history and obtains vitals while a physician documents relevant findings and enters orders as diagnostic and other needs emerge. |  | MA Skill Development | Increased health measures (cancer screening) | Lower wait time; more efficient visits (e.g. better communication) |  |

**Table 2. Training or credentials for MOA (8 articles)**

|  |  | Impact on the Quintuple Aim | | | | |
| --- | --- | --- | --- | --- | --- | --- |
| Author (Year)  Country | Intervention details | Patient/Care Experience | Workforce Well-Being | Population Health | Cost of Care/ Efficiency | Equity |
| Chapman (2017)  **USA** | Training and compensation for new MA roles: health coach, medical scribe, dual role translator, health navigator, panel manager, cross-trained flexible role, and supervisor. | Patient satisfaction | Staff satisfaction and engagement with training | Clinical screening of health outcomes | Increased visit time; MA retention; Less urgent care and hospital visits; Increased phone call returns |  |
| Gray (2021)  **USA** | **MAs were trained to take part in team-based care. Including:** chronic disease management; health coaching patient activation strategies; discussed and document self-management care plan; discussed community resources |  | MA confidence, motivation, and leadership in their work |  |  |  |
| Brown, (2013)  **USA** | MA mental health communication training to encourage families to discuss mental health problems with primary care providers.  **(e.g., body language, explaining their role and next steps, asking open-ended questions, acknowledging concerns)** | Positive perceptions of MAs ;  Patient communication; Patient comfort discussing mental health; | MA engagement in training; Uptake of skills |  | Visit Length |  |
| Jopson (2022)  **USA** | MA apprenticeship program (e.g., taking vitals, preliminary health screenings, and managing prescription refills). |  | Creation of career pathways and opportunities; recruitment of MAs; job satisfaction and engagement; fostered a culture of teaching and learning among staff and providers |  |  |  |
| Blash (2011)  **USA** | Trainers and administrators worked to develop a Medication Safety training program for certified MAs. |  |  |  |  |  |
| Blash (2011)  **USA** | Training for MAs in community health centers to allow them to serve as medical interpreter, patient navigator, and community health worker while providing career ladder opportunities. |  | MA Recruitment and Retention; Provider and MA Satisfaction with work; MA Skill building; |  |  | Priority for bilingual MAs |
| Blash (2011)  **USA** | The Administrative Support Training Program was a 6-to-8-week training and on-boarding session for new clerical staff. | Patient satisfaction with care | Workforce Commitment;  Staff retention;  Employee Satisfaction | Immunization rates and colorectal screening | Employee benefits;  Less ER/ specialty care visits |  |
| Vadala (2021), **Canada** | This book introduces the role of the health services administrator within the broader health care system. |  |  |  |  |  |

**Table A5. Team building or reconfiguration of the team (16 articles)**

|  |  | Impact on the Quintuple Aim | | | | |
| --- | --- | --- | --- | --- | --- | --- |
| Author (Year) | Intervention details | Patient/Care Experience | Workforce Well-Being | Population Health | Cost of Care/ Efficiency | Equity |
| Dill, J. (2021), USA | Use of **career ladders for MAs** in primary care practices as a mechanism for increasing wages and career opportunity. |  | Increased motivation and incentive for MAs; Wage increases and career opportunities |  |  |  |
| Eden (2016)  **USA** | **New care delivery model: delegated** tasks to MAs, (e.g., managing lab and radiology results, informing patients of results, documenting complaints and vital signs, reviewing preventive services and providing referrals) | Patient satisfaction with care | Provider satisfaction,  MA satisfaction and engagement with roles | Patient visits per day;  Increased patient contact |  |  |
| Gerstein (2015)  **USA** | T**eam-based primary care with MAs:** formally assigning all patient-facing staff to teams, assigning patient panel to each team, daily team huddles and regular team meetings, assigning MD-specific tasks to MAs, calling patients for preventative care screening. |  | MA fulfilment in their work ; increased tolerance of workload |  |  |  |
| Dill, J. (2021), USA | **Patient-centered medical home model** included higher skill job tasks (e.g. health coaching, panel management, EHR documentation support), hiring highly trained MAs, creating formal partnerships with MA training institutions and a career ladder. |  |  |  |  |  |
| Ferrante, J. M. (2018), USA | **The Patient-centered medical home model** included pre-visit planning, patient education materials, conduct awareness campaigns and document screenings |  | MA Job satisfaction |  |  |  |
| Brant, H. D. (2018), United Kingdom | **Alternatives to face-to-face consultation** (telephone, email, e-consultation, internet video) with an increased role for receptionists. This includes **offering the option of e-consultations/telephone calls to patients over in-person appts.** |  | MA attitudes towards workload; Low uptake of the intervention due to lack of involvement in planning, training, acceptability, and support for MOAs |  | Cost-effective for the practice |  |
| Brooks, J. V. (2020), USA | The **'Collaborative Resident MA Dynamic' model (team-based care)** involved **task switching and blurred roles** between MAs and medical residents. MAs also shared their knowledge about clinic practices to medical residents. |  | MA empowerment and leadership; Increase in communication; Task switching/coordination of work |  |  |  |
| Milford, J. (2018), USA | Increased ratio of MAs to physicians from 1:1 to 3:2, and trained MAs for expanded roles during exam-room entry and discharge, including **assisting with documentation** during the patient visit, **coordinating patient handoffs, record histories and exam results, treatment plans, print visit summary**, etc. | Patient gratitude | Employee satisfaction |  |  |  |
| Martinez, M (2014), USA | A **work performance feedback form "shareport"** cards allowed physicians to provide feedback on MA’s performance in the clinic. Helped physicians and MAs make time for **meetings** to discuss the feedback form. | Patient satisfaction scores | Physician satisfaction; Staff satisfaction |  | More staff time for pre-clinic work; Physicians complete clinic work earlier |  |
| Grove (2010), USA | This intervention wanted to identify and eliminate waste activities through use of clinical staff and tools like value-stream mapping. |  |  |  | More time spend on valuable activities |  |
| Johnson, Paula A. (2011), USA | Physicians and staff members were involved in assessing and redesigning their work environment, and new practices were developed to e**nhance patient care and improve work environment.** |  | Skill building; Collaboration;  Distributed leadership; problem solving mindset; Enhanced employee voices; Flexible work;  Shift from individual to a team approach |  |  |  |
| Blash (2011), USA | A quality assurance committee made up of providers, administrators, clinical staff and board members to monitor and evaluate outcomes, including those related to efficiency, access, patient satisfaction, administrative processes and clinical outcomes using the **Chronic Care Model.** | Patient satisfaction | Provider satisfaction;  Recruitment and Retention | Diabetic care;  Childhood immunization;  heart / stroke measures (CVD diagnosis) | decreased the number of abandoned  calls |  |
| Blash (2011), USA | **Cross-training** that allowed **MAs to learn laboratory, front desk, or medical records skills.** Career ladder was also built. |  | High retention of staff;  MA job satisfaction |  |  |  |
| Blash (2011), USA | The **Team-Based Model of Care** with a ratio of 5 MOAs:2 physicians. Thereby provided each patient an **“ambassador” who could guide patients through the visit and** taking on physician tasks. | patient satisfaction | provider satisfaction;  MA recruitment and retention maintained | increase in vaccinations,  diabetes measures, testing for HbA1C and rate of LdL and Breast screening | Decreased patient no shows; visit cycle times; and costs decreased; increased profit;  Staff:patient ratio decreased |  |
| Kwan (2022) **USA** | Primary care redesign: included rapid improvement events, changing clinic space configurations, performance dashboards, and practice coaching to expand MA roles. |  | MA confidence and skill building; change in workflow |  | Staffing challenges |  |
| Blash (2011), USA | Expanded role of medical assistants by providing them with additional training and responsibility: performing vitals, providing educational materials and tracking follow ups. | Patient satisfaction | Improved Teamwork; Increased career ladder opportunities + pay raise, benefits; Less staff turnover; Staff satisfaction |  | Shorter wait times; fewer calls + better communication; cost savings on staff |  |
| DeChant (2021), USA | A toolkit to understand the current dividers of physician-administrator distrust and how this contributes to burnout. |  |  |  |  |  |
| Lyon (2022), USA | These strategies for recruiting and retaining staff can help medical practices survive the “Great Resignation.” |  |  |  |  |  |
